# Modeling a pesticide remediation strategy for preparative liquid chromatography using high-performance liquid chromatography

**DOI:** 10.1186/s42238-023-00172-1

**Published:** 2023-04-13

**Authors:** Jamie Cuchiaro, James Baumgartner, Melissa M. Reynolds

**Affiliations:** 1 Department of Chemistry, Colorado State University, 1872 Campus Delivery, CO 80523 Fort Collins, USA; 2 Panacea Life Sciences, 16194 W 45th Dr, CO 80403 Golden, USA; 3 School of Biomedical Engineering, Colorado State University, 1376 Campus Delivery, 80523 Fort Collins, CO USA; 4 Department of Chemical and Biological Engineering, Colorado State University, 1370 Campus Delivery, CO 80523 Fort Collins, USA

**Keywords:** Cannabis, Cannabinoids, Pesticide remediation, Adsorptive separation, High-performance liquid chromatography, Preparative Liquid Chromatography

## Abstract

**Background:**

*Cannabis sativa L.* also known as industrial hemp, is primarily cultivated as source material for cannabinoids cannabidiol (CBD) and ∆9-tetrahydrocannabinol (∆9-THC). Pesticide contamination during plant growth is a common issue in the cannabis industry which can render plant biomass and products made from contaminated material unusable. Remediation strategies to ensure safety compliance are vital to the industry, and special consideration should be given to methods that are non-destructive to concomitant cannabinoids. Preparative liquid chromatography (PLC) is an attractive strategy for remediating pesticide contaminants while also facilitating targeted isolation cannabinoids in cannabis biomass.

**Methods:**

The present study evaluated the benchtop-scale suitability of pesticide remediation by liquid chromatographic eluent fractionation, by comparing retention times of 11 pesticides relative to 26 cannabinoids. The ten pesticides evaluated for retention times are clothianidin, imidacloprid, piperonyl butoxide, pyrethrins (I/II mixture), diuron, permethrin, boscalid, carbaryl, spinosyn A, and myclobutanil. Analytes were separated prior to quantification on an Agilent Infinity II 1260 high performance liquid chromatography with diode array detection (HPLC-DAD). The detection wavelengths used were 208, 220, 230, and 240 nm. Primary studies were performed using an Agilent InfinityLab Poroshell 120 EC-C18 3.0 × 50 mm column with 2.7 μm particle diameter, using a binary gradient. Preliminary studies on Phenomenex Luna 10 μm C18 PREP stationary phase were performed using a 150 × 4.6 mm column.

**Results:**

The retention times of standards and cannabis matrices were evaluated. The matrices used were raw cannabis flower, ethanol crude extract, CO_2_ crude extract, distillate, distillation mother liquors, and distillation bottoms. The pesticides clothianidin, imidacloprid, carbaryl, diuron, spinosyn A, and myclobutanil eluted in the first 3.6 min, and all cannabinoids (except for 7-OH-CBD) eluted in the final 12.6 min of the 19-minute gradient for all matrices evaluated. The elution times of 7-OH-CBD and boscalid were 3.44 and 3.55 min, respectively.

**Discussion:**

7-OH-CBD is a metabolite of CBD and was not observed in the cannabis matrices evaluated. Thus, the present method is suitable for separating 7/11 pesticides and 25/26 cannabinoids tested in the six cannabis matrices tested. 7-OH-CBD, pyrethrins I and II (RT_A_: 6.8 min, RT_B_: 10.5 min), permethrin (RT_A_: 11.9 min, RT_B_: 12.2 min), and piperonyl butoxide (RT_A_: 8.3 min, RT_B_: 11.7 min), will require additional fractionation or purification steps.

**Conclusions:**

The benchtop method was demonstrated have congruent elution profiles using preparative-scale stationary phase. The resolution of pesticides from cannabinoids in this method indicates that eluent fractionation is a highly attractive industrial solution for pesticide remediation of contaminated cannabis materials and targeted isolation of cannabinoids.

## Background

**Fig. 1:**
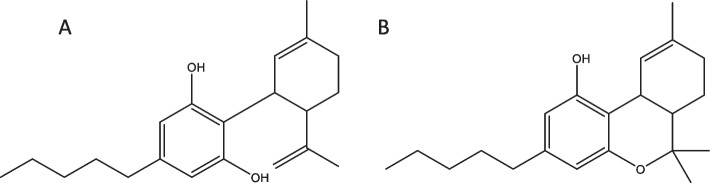
Molecular structures of **A **CBD and **B **∆9-THC, two high value cannabinoids that are produced in high abundance in the cannabis plant

*Cannabis sativa L.*(cannabis) has been used for over 6000 years for medicinal, utilitarian, recreational, and religious purposes (Bonini et al. 2018; Luca et al. 2020). Cannabis produces a number of pharmacoactive compounds, including phytocannabinoids, terpenoids, flavonoids, and alkaloids (Brenneisen 2007). Phytocannabinoids are terpenophenolic molecules; perhaps the best known are ∆9-tetrahydrocannabinol (∆9-THC) and the non-psychoactive cannabidiol (CBD) (Citti et al. 2016). THC produces the recreational “high” associated with marijuana, and CBD is a non-psychoactive analog that is reported to have antiemetic, anti-seizure, and anti-inflammatory properties (Sandler et al. 2019). C. sativa can be broadly differentiated into two types: hemp and recreational or medicinal marijuana (McPartland and Guy 2017; Mudge et al. 2017). The distinction between marijuana and hemp is defined by the relative THC content on the dry weight basis. Hemp is characterized as having less than 0.3% (w/w) THC, whereas marijuana has THC in amounts above that threshold (Rustichelli et al. 1998; Small and Beckstead 1973; Hazekamp and Fischedick 2012). In general, hemp produces CBD in much greater amounts than marijuana, and is thus an important cash crop in the emerging cannabis industry (Nie et al. 2019). The molecular structures of ∆9-THC and CBD are shown in Fig. 1.

**Fig. 2:**
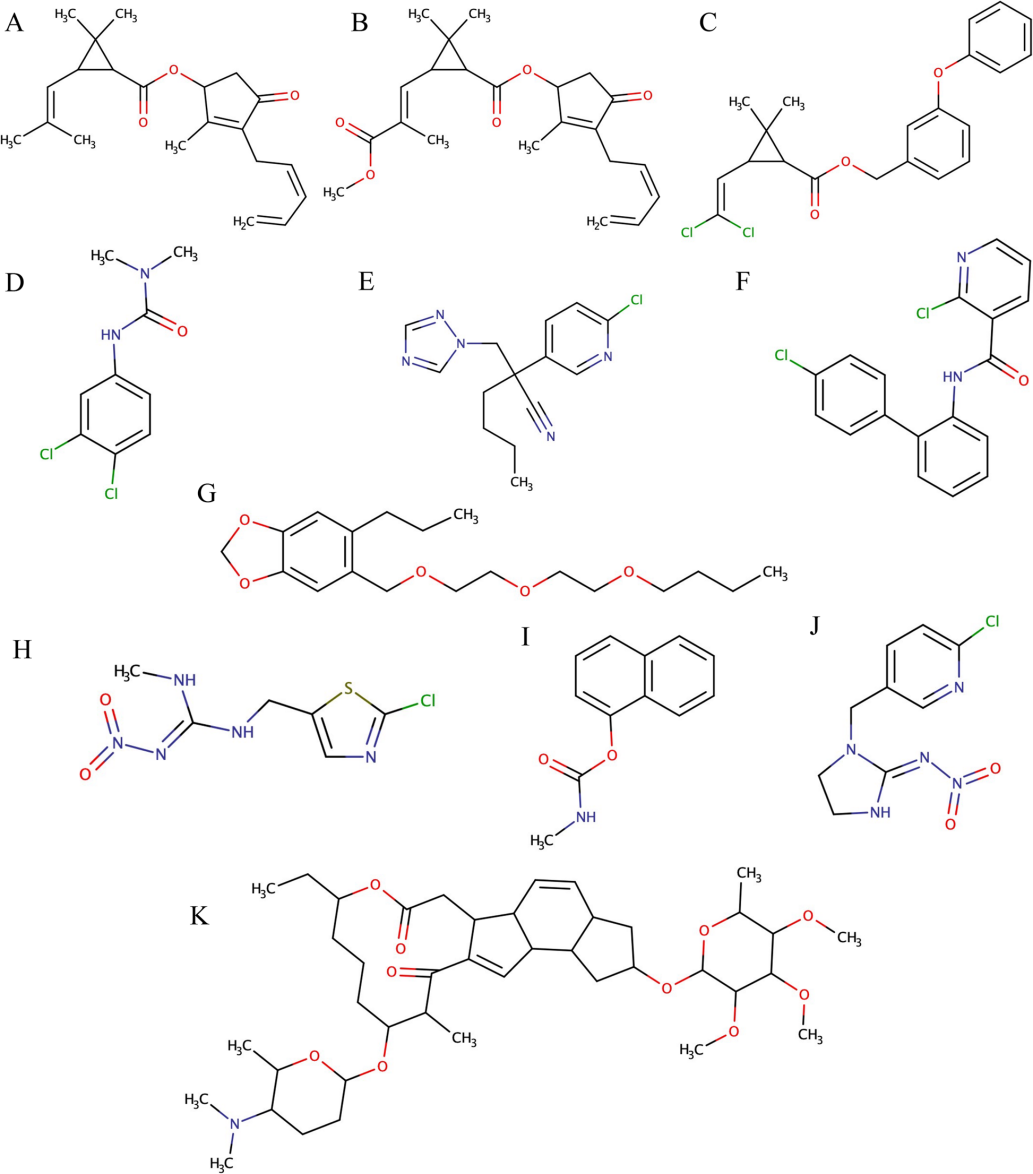
Structures of pesticides investigated for the present HPLC-QQQ method. **A** pyrethrin I, **B** pyrethrin II, **C** permethrin, **D** diuron, **E** myclobutanil, **F** boscalid, **G** piperonyl butoxide, **H** clothianidin, **I** carbaryl, **J** imidacloprid, **K** Spinosyn A

Pesticide contamination of cannabis source material is a primary concern in the production of cannabis products, and acceptability criteria for the presence of pesticides are regulated by state agencies (Subritzky et al. 2017). Although direct use of pesticides on hemp products is regulated, occasionally the plants are indirectly exposed to banned pesticides. Cannabis is a hyperaccumulator; therefore, trace contamination of biomass is an issue when grown next to other crops (McPartland and McKernan 2017; Wu et al. 2021). For example, hemp grown in proximity to other commodity crops can be contaminated by pesticides from those adjacent fields (López-Ruiz et al. 2022). This becomes problematic when the pesticides used are not accepted by commercial cannabis regulations and drift to the hemp crop. A representative group of 11 banned pesticides was selected for use in this study, comprised of carbaryl, boscalid, spinosyn A, imidacloprid, clothianidin, diuron, myclobutanil, piperonyl butoxide, pyrethrins I and II, and permethrin (Pesticide 2022). Their structures are presented in Fig. 2.

Carbaryl, boscalid, spinosyn A, imidacloprid, clothianidin, diuron, myclobutanil, piperonyl butoxide, pyrethrins I and II, and permethrin are all pesticides banned from use in cannabis products (Pesticide 2022). Their high chemical and thermal stability make them difficult to remediate from contaminated plant and extract materials, and remediation is further complicated by the complex nature of the cannabis matrix (Wylie et al. 2020; do Amaral et al. 2022). These 11 pesticides are ideal contaminants for demonstrating proof-of-concept of a liquid chromatographic method for remediating pesticides at the benchtop scale, because of their varied chemical composition and unsuitability for cannabis material (Craven et al. 2019).

Cannabis products intended for consumption must be tested to ensure compliance with state regulatory agencies; products with pesticide content exceeding acceptability criteria must be remediated prior to sale (Subritzky et al. 2017; Marijuana 2020; Geesaman n.d. ). PLC is an attractive remediation strategy that separates sample components using a liquid-solid interface, where solvent composition is modulated as sample moves through a column, and operates on the same principles as analytical High-Performance Liquid Chromatography (HPLC) (De Luca et al. 2022). The purpose of this study was to develop a benchtop-scale HPLC method for separating 11 pesticides and 26 cannabinoids, and evaluate the theoretical suitability of the method for separating pesticides and cannabinoids from six industrial hemp processing matrices using preparative-scale liquid chromatography (PLC). Methods for both cannabinoid and pesticide quantitation have been reported using HPLC, but targeted pesticide separation and assay for scale-up to PLC has not been reported (Craven et al. 2019; Atapattu and Johnson 2020; López-Ruiz et al. 2022). Using a benchtop-scale HPLC adds an element of novelty to this work by incorporating principles of green chemistry for developing preparative-scale liquid chromatography methods of interest to the broader industrial community.

Ideal pesticide remediation methods also maximize cannabinoid recovery in tandem, since cannabinoids are also present in sample matrices (Moulins et al. 2018). Suitability of the present method for each pesticide was evaluated based on observed retention time. A shorter retention time than that of the cannabinoids in matrix indicates that the method is suitable, whereas pesticides with retention times longer than cannabinoids would require additional separation steps. Pesticides were evaluated as single samples and retention times measured. The suitability of benchtop-scale method on six processing matrices was evaluated: flower, CO_2_crude extract, ethanol crude extract, distillate, distillation mother liquor, and distillation bottoms. These matrices were selected as representative steps in industrial processing based on recommendation from Panacea Life Sciences. Flower, crude extracts, and distillates having the most direct path to commercial sale, and the distillation mother liquor and bottoms are processing side-products with potentially commercializable components but require additional processing to recover (Gould and The Cannabis Crop 2015; Aizpurua-Olaizola et al. 2014; King 2019).

A limitation of the results reported in "Demonstration of congruent separations on 10 μm luna C18" section is that analytes were separated on a column with Agilent Poroshell 120 2.7 μm C18 media (Neue 1997). Column dimensions and packing density are different between benchtop and preparative scale columns, but particle size is a key consideration in stationary phase selection (Bączek et al. 2005; Altiero 2018). 2.7 μm Poroshell stationary phase is not available for bulk purchase and cannot be used for PLC scale up. The stationary phase for future preparative-scale separations indicated by this study will be Phenomenex Luna 10 μm C18 PREP media. Preliminary experiments have been performed on a 150 × 4.6 mm Phenomenex Luna 10 μm C18 PREP column to better evaluate suitability of the method. The larger particle size of Luna PREP media is expected to decrease operating pressure but also decrease method resolution (Molnár 2005; Neue and Kele 2007; Morley and Minceva 2020). Lower resolution is not a critical concern for the proposed preparative method, as long as relative retention times between pesticides and cannabinoids are sufficiently different. Preliminary results collected on the 150 × 4.6 mm 10 μm Luna column are reported to demonstrate congruence between the two packing media.

## Methods

### Materials

HPLC-grade acetonitrile (PN A998-4) was purchased from Fisher Scientific. Carbaryl (PN 24,139), boscalid (PN 24,135), spinosyn (PN 25,649), diuron (PN 24,040), myclobutanil (PN 24,100), clothianidin (PN 29,605), pyrethrins I/II (PN 25,814), piperonyl butoxide (PN 25,820), imidacloprid (PN 24,130), and permethrin (PN 23,821) were purchased from Cayman Chemical, Ann Arbor, Michigan. EN Method 15,662 (QuECHERS, PN 5982 − 5650) salts were purchased from Agilent Technologies, Santa Clara, California. A Sigma Millipore Direct-Q 5 water filtration system was used to deliver 18.0 mΩ•cm water. Cannabis matrix samples were donated by Panacea Life Sciences, Golden, CO.

Reference materials of ∆8-tetrahydrocannabinol (∆8-THC; PN: ISO60158), ∆9-tetrahydrocannabinol (∆9-THC, PN: ISO60157), ∆9-Tetrahydrocannabinolic acid (∆9-THCA; PN: 33,448), ∆9-tetrahydrocannabutol (∆9-THCB; PN: 33,078), ∆9-tetrahydrocannabihexol (∆9-THCH; PN: 33,352), ∆9-tetrahydrocannabiphorol (∆9-THCP; PN: 30,171), ∆9-tetrahydrocannabivarin (∆9-THCV; PN: 18,091), ∆9-tetrahydrocannabivarinic acid (∆9-THCVA; PN: 21,259), 7-OH-cannabidiol (7-OH-CBD; PN: 36,517), cannabichromene (CBC; PN: 26,252), cannabichromeorcin (CBCO; PN: 21,742), cannabichromevarin (CBCV; PN: 21,974), cannabichromevarinic acid (CBCVA; PN: 32,718), cannabidiol (CBD; PN: 21,259), cannabidiolic acid (CBDA; PN: 18,090), cannabidiolic acid methyl ester (CBDA-ME; PN: 28,347), cannabidiphorol (CBDP; PN: 30,169 ), cannabidivarin (CBDV; PN: 20,165), cannabielsoin (CBE; PN: 21,092), cannabigerol (CBG, PN: 20,164), cannabigerolic acid (CBGA PN: 20,019), cannabigerol quinone acid (CBGAQ, PN: 31,772), cannabigerovarin (CBGV PN: 29,117), cannabigerovarinic acid (CBGVA; PN: 25,469), cannabicyclol (CBL; PN: 22,036), cannabinol (CBN; PN: 25,495), cannabicitran (CBT; PN: 21,295), and olivetol (PN: 35,202) were purchased from Cayman Chemical, Ann Arbor, Michigan.

### Standard preparation

All standards were prepared at ambient temperatures and stored at -20 °C. Stock standard of 250 µg/mL per pesticide was prepared in acetonitrile. The working standard was prepared by diluting the stock 1:3 in acetonitrile. Calibration standards were prepared by dilution of the working standard and contained 25% matrix blank. Retention time check standards were prepared by dilution of CRMs in 10.0 mL class A volumetric flasks to nominal concentrations of 100 ug/mL.

### Sample preparation

A mass of 100 ± 10 mg of sample were massed into 50 mL falcon tubes, soaked in 10 mL water, and vortexed. The same mass was used for all matrices evaluated. Then, 10 mL acetonitrile, 4 g MgSO_4_, 1 g NaCl, 0.5 g disodium citrate sesquihydrate, 1 g sodium citrate, and ceramic agitator were added to the samples. Samples were then rigorously vortexed for 1 min, centrifuged, and the acetonitrile (top) layer was decanted and transferred into 1.5 mL HPLC vials. Samples were prepared in triplicate.

### Instrument method

Analytes were separated prior to quantification on an Agilent Infinity II 1260 high performance liquid chromatography with diode array detection (HPLC-DAD). The detection wavelengths used were 208, 220, 230, and 240 nm. Primary studies were performed using an Agilent InfinityLab Poroshell 120 EC-C18 3.0 × 50 mm column with 2.7 μm particle diameter (PN: 699975-302), using a binary gradient. Preliminary studies on Phenomenex Luna 10 μm C18 PREP stationary phase were performed using a 150 × 4.6 mm column (PN: 00G-4616-E0). Mobile phase A was 0.1% (v/v) phosphoric in water. Mobile phase B was 0.1% (v/v) phosphoric acid in acetonitrile. The injection volume was 1 µL. The method gradient was 60% B, 0 min; 60% B, 1 min; 80% B, 8 min; 100% B, 10 min; 100%, 14.5 min; 60% B, 15 min; 19 min runtime. The method was calibrated using the calibration standard solutions (R^2^ > 0.999, all cases).

Measurements were quantitated using an external standard calibration. The instrument signal used was defined as the integrated peak area in the DAD chromatogram with units of absorbance over time (mAU*s).

## Results and discussion

### Analyte retention on 2.7 μm Poroshell C18

As expected, carbaryl, boscalid, and spinosyn A eluted before the more hydrophobic cannabinoids during spike/recovery trials. Clothianidin, imidacloprid, carbaryl, olivetol, diuron, spinosyn A, boscalid, and myclobutanil eluted in the first 3.6 min, and all cannabinoids (except for 7-OH-CBD) eluted in the final 12.6 min of the 19-minute gradient for all matrices evaluated. The retention time of 7-OH-CBD (3.441 min) is reported in this work although is a metabolite of CBD and therefore not expected in cannabis matrices. Thus, the present method is suitable for 7/11 pesticides and 25/26 cannabinoids evaluated, by fractionating the first 19% of eluent to waste. 7-OH-CBD, and the pesticides pyrethrins I and II, permethrin, and piperonyl butoxide will require additional purification or fraction collection steps following the present gradient. Retention times for all analytes are shown in Table 1 and organized both alphabetically and by elution time.

**Table 1:** Elution times for all analytes, organized alphabetically and by elution time. Clothianidin, imidacloprid, carbaryl, diuron, spinosyn A, and myclobutanil elute before all cannabinoids and the present method is suitable for remediating them by fractionating the first 19% of eluent to waste. Retention times were set at ± 2%

	*Analyte (Alphabetical)*	*Retention Time (min)*	*Analyte* *(Elution order)*	*Retention Time (min)*
1	∆8-THC	11.510	Clothianidin	1.451
2	∆9-THC	11.610	Imidacloprid	1.492
3	∆9-THCA	12.320	Carbaryl	2.160
4	∆9-THCB	10.678	Diuron	2.314
5	∆9-THCH	12.331	Spinosyn A	2.480
6	∆9-THCP	12.915	Myclobutanil	3.258
7	∆9-THCV	9.160	7-OH-CBD	3.441
8	∆9-THCVA	10.646	Boscalid	3.550
9	7-OH-CBD	3.441	CBGV	6.440
10	Boscalid	3.550	CBDV	6.490
11	Carbaryl	2.160	CBGVA	6.500
12	CBC	12.180	Pyrethrin Peak A	6.829
13	CBCO	7.866	CBGQA	7.136
14	CBCVA	11.224	CBE	7.636
15	CBD	8.900	CBCO	7.866
16	CBDA	8.000	CBDA	8.000
17	CBDA-ME	12.314	Pip. But. Peak A	8.327
18	CBDP	11.269	CBGA	8.340
19	CBDV	6.490	CBG	8.704
20	CBE	7.636	CBD	8.900
21	CBG	8.704	THCV	9.160
22	CBGA	8.340	Pyrethrin Peak B	10.480
23	CBGQA	7.136	THCVA	10.646
24	CBGV	6.440	∆9-THCB	10.678
25	CBGVA	6.500	CBN	10.900
26	CBL	12.098	CBCVA	11.224
27	CBN	10.900	CBDP	11.269
28	CBTC	13.178	∆8-THC	11.510
29	Clothianidin	1.451	∆9-THC	11.610
30	Diuron	2.314	Pip. But. Peak B	11.748
31	Imidacloprid	1.492	Permethrin Peak A	11.880
32	Myclobutanil	3.258	CBL	12.098
33	Permethrin Peak A	11.880	Permethrin Peak B	12.175
34	Permethrin Peak B	12.175	CBC	12.180
35	Pip. But. Peak A	8.327	CBDA-ME	12.314
36	Pip. But. Peak B	11.748	∆9-THCA	12.320
37	Pyrethrin Peak A	6.829	∆9-THCH	12.331
38	Pyrethrin Peak B	10.480	∆9-THCP	12.915
39	Spinosyn A	2.480	CBTC	13.178

Multiple peaks were observed in Pyrethrins, piperonyl butoxide, and permethrin standards. Piperonyl butoxide is sold as a ≥ 95% liquid and permethrin is sold as a 95% solid formulation, and it is expected that additional peak is a formulation impurity. Pyrethrin is sold as a mixture of pyrethrins I and II. For cases where multiple peaks were observed, peaks were identified as A and B because their identities could not be deduced from the DAD data alone. It is hypothesized that pyrethrin and piperonyl butoxide peak identities may be elucidated using mass spectrometric methods but this falls outside the scope of the present paper.

### Demonstration of congruent separations on 10 μm luna C18

**Fig. 3:**
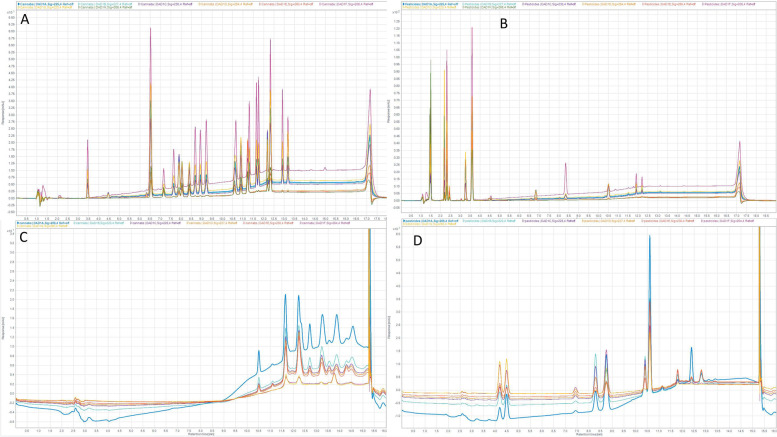
Representative chromatograms of cannabinoid (**A** and **C**) and pesticide (**B** and **D**) test samples. Chromatograms **A** and **B** used the instrument method reported in "Instrument method" section. The large peak occurring at 11.1 min is likely unresolved late-eluting species such as CBTC and permethrin. Chromatograms **C** and **D** used a flow rate of 1.0 mL/min, causing peaks to elute earlier

Preliminary injections using larger particle size were done using 11-component pesticide, and 26-component cannabinoid mixtures. All components were evaluated, including those deemed unsuitable on the 2.7 μm column, for comparison between the two stationary phases. Analyte concentrations and scale were not normalized which causes the difference in peak size. Pesticide and cannabinoid elution profiles are congruent to those in "Analyte retention on 2.7 µm Poroshell C18" section, in that cannabinoids elute late in the gradient, more polar pesticides elute earlier in the gradient, and some pesticides peaks overlap with cannabinoids. Representative chromatograms are shown in Fig. 3. Chromatograms A and B used the instrument method reported in section. The solvent front and first eluting species are shifted right in the chromatogram, caused by the 5 cm longer pathlength through the column. The large peak occurring at 11.1 min is likely unresolved late-eluting species such as CBTC and permethrin. Chromatograms C and D used a flow rate of 1.0 mL/min, causing shifts in retention times.

### Matrix cannabinoid profiles

**Fig. 4:**
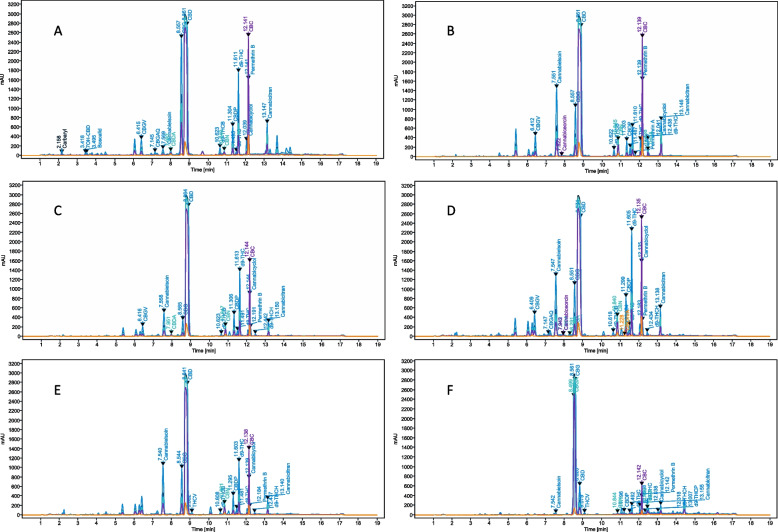
Representative chromatograms of **A** raw flower, **B** crude CO_2_ extract, **C** crude ethanol extract, **D** CBD distillate, **E** mother liquor, and **F** distillation bottoms. Each matrix sample was spiked with carbaryl, boscalid, and spinosyn A for reference. The ratio of CBDA to CBD occurs in greatest proportion in the raw flower sample, because the biomass is decarboxylated with heat prior to crude extraction. CBD/CBDA was observed in greatest relative abundance for each of the matrices except the distillation bottoms, where CBG occurs in greatest abundance

Cannabinoids were qualitatively identified in each of the spiked hemp matrices. The significance being that targeted isolation of cannabinoids following pesticide remediation is possible using PLC. The same mass of each matrix was used during sample preparation, although industrial preparations will scale separately due to the different relative concentrations of cannabinoids in each matrix. Raw flower has a smaller percent composition on a dry weight basis than extracts or distillates. Targeted isolation can be optimized by matrix selection, based on the qualitative profiles. It was expected that the ratio of acid-form to decarboxylated cannabinoids will be observed in greatest proportion in the raw flower sample, because the plant biomass is decarboxylated by heating prior to crude extraction. CBD/CBDA was observed in greatest relative abundance for each of the matrices except the distillation bottoms, where CBG occurs in greatest abundance. Representative chromatograms for each matrix are shown in Fig. 4.

## Conclusion

The present study evaluated retention times of 11 pesticides relative to 26 cannabinoids, for general suitability of remediation by eluent fractionation with PLC. Six industrial cannabis processing matrices were used: flower, ethanol crude extract, CO_2_ crude extract, distillate, distillation mother liquor, and distillation bottoms. Clothianidin, imidacloprid, carbaryl, diuron, spinosyn, boscalid, and myclobutanil eluted in the first 3.6 min, and all cannabinoids (except for 7-OH-CBD) eluted in the final 12.6 min of the 19-minute gradient for all matrices evaluated. 7-OH-CBD is a metabolite of CBD and is not expected in cannabis extracts. Thus, the present method is suitable for simple fractionation of 7/11 pesticides and 25/26 cannabinoids evaluated on 2.7 μm C18 Poroshell media. 7-OH-CBD, pyrethrins I and II, permethrin, and piperonyl butoxide will require additional purification beyond the present gradient. Complimentary injections were made on 10 μm C18 PREP stationary phase and the elution profiles were congruent to those on the 2.7 μm media. The possibility of completely removing pesticides while retaining cannabinoids and other high-value matrix components for further processing makes PLC a highly attractive strategy for separating cannabinoids in large volume and industrial manufacturing of cannabis products.

## Data Availability

The data sets used and/or analyzed during this study are available from the corresponding author upon reasonable request.

## Abbreviations

ACN: Acetonitrile
HPLC: High performance liquid chromatography
DAD: Diode array detection
CBD: Cannabidiol
CBN: Cannabinol
THC: ∆-9-tetrahydrocannabinol
MeOH: Methanol
CO2: Carbon dioxide
EtOH: Ethanol
CBDA: Cannabidiolic acid
CBG: Cannabigerol
CBTC: Cannabicitran
∆8-THC: ∆8-tetrahydrocannabinol
∆9-THCA: ∆9-tetrahydrocannabinolic acid
∆9-THCB: ∆9-tetrahydrocannabutol
∆9-THCH: ∆9-tetrahydrocannabihexol
∆9-THCP: ∆9-tetrahydrocannabiphorol
∆9-THCV: ∆9-tetrahydrocannabivarin
∆9-THCVA: ∆9-tetrahydrocannabivarinic acid
7-OH-CBD: 7-hydroxy-cannabidiol
CBC: Cannabichromene
CBCO: Cannabichromeorcin
CBCVA: Cannabichromivarinic acid
CBDA-ME: Cannabidiolic acid methyl ester
CBDP: Cannabidiphorol
CBDV: Cannabidivarin
CBE: Cannabielsoin
CBGA: Cannabigerolic acid
CBGQA: Cannabigerol quinone acid
CBGV: Cannabigerovarin
CBGVA: Cannabigerovarinic acid
CBL: Cannabicyclol
